# Ketone Body Exposure of Cardiomyocytes Impairs Insulin Sensitivity and Contractile Function through Vacuolar-Type H^+^-ATPase Disassembly—Rescue by Specific Amino Acid Supplementation

**DOI:** 10.3390/ijms232112909

**Published:** 2022-10-26

**Authors:** Shujin Wang, Dietbert Neumann, B. Daan Westenbrink, Francesco Schianchi, Li-Yen Wong, Aomin Sun, Agnieszka Strzelecka, Jan F. C. Glatz, Joost J. F. P. Luiken, Miranda Nabben

**Affiliations:** 1 Department of Genetics and Cell Biology, Faculty of Health, Medicine and Life Sciences, Maastricht University, 6200 MD Maastricht, The Netherlands; 2 Institute of Life Sciences, Chongqing Medical University, Chongqing 400032, China; 3 Department of Pathology, Maastricht University Medical Center^+^, 6200 MD Maastricht, The Netherlands; 4 CARIM School for Cardiovascular Diseases, 6229 ER Maastricht, The Netherlands; 5 Department of Cardiology, University of Groningen, University Medical Center Groningen, P.O. Box 30.001, 9700 RB Groningen, The Netherlands; 6 Departments of Clinical Genetics, Maastricht University Medical Center^+^, 6200 MD Maastricht, The Netherlands

**Keywords:** ketone bodies, vacuolar-type H+-ATPase, endosomal CD36, lipid-induced insulin resistance, contractile function, diabetic heart

## Abstract

The heart is metabolically flexible. Under physiological conditions, it mainly uses lipids and glucose as energy substrates. In uncontrolled diabetes, the heart switches towards predominant lipid utilization, which over time is detrimental to cardiac function. Additionally, diabetes is accompanied by high plasma ketone levels and increased utilization of energy provision. The administration of exogenous ketones is currently being investigated for the treatment of cardiovascular disease. Yet, it remains unclear whether increased cardiac ketone utilization is beneficial or detrimental to cardiac functioning. The mechanism of lipid-induced cardiac dysfunction includes disassembly of the endosomal proton pump (named vacuolar-type H+-ATPase; v-ATPase) as the main early onset event, followed by endosomal de-acidification/dysfunction. The de-acidified endosomes can no longer serve as a storage compartment for lipid transporter CD36, which then translocates to the sarcolemma to induce lipid accumulation, insulin resistance, and contractile dysfunction. Lipid-induced v-ATPase disassembly is counteracted by the supply of specific amino acids. Here, we tested the effect of ketone bodies on v-ATPase assembly status and regulation of lipid uptake in rodent/human cardiomyocytes. 3-β-hydroxybutyrate (3HB) exposure induced v-ATPase disassembly and the entire cascade of events leading to contractile dysfunction and insulin resistance, similar to conditions of lipid oversupply. Acetoacetate addition did not induce v-ATPase dysfunction. The negative effects of 3HB could be prevented by addition of specific amino acids. Hence, in sedentary/prediabetic subjects ketone bodies should be used with caution because of possible aggravation of cardiac insulin resistance and further loss of cardiac function. When these latter maladaptive conditions would occur, specific amino acids could potentially be a treatment option.

## 1. Introduction

The heart is a metabolically flexible organ that can use a variety of substrates for energy provision. To fulfill cardiac energy requirements, the healthy adult heart mainly uses fatty acids (FA) and glucose, but it will switch its substrate preference to alternative substrates such as ketone bodies and amino acids (AA) when being exposed to physiological or pathological stimuli [1,2]. Ketone bodies are synthesized predominantly in the liver under conditions where there is a limited carbohydrate and surplus fatty acid availability [3]. Ketone bodies are synthesized from FA oxidation (FAO)-derived acetyl-coenzyme A, and then are transported to key extrahepatic oxidative tissues such as the heart and brain for oxidation [4,5]. Myocardial ketone body delivery is particularly increased under physiological conditions such as fasting, starvation, post-exercise, the neonatal period, pregnancy, and also during pathological conditions, such as uncontrolled diabetes [6,7,8]. The circulating concentration of ketone bodies in healthy individuals normally exhibits circadian oscillations between around 100 µM and 250 μM but can rise to ∼1 mM after prolonged exercise, 5–7 mM after prolonged fasting, and as high as 20 mM in pathological states (e.g., untreated insulin-deficient diabetes) [7,9]. The increase in plasma ketone body levels is suggested to be an adaptive and compensatory response to an energy deficit [7].

Dietary supplementation of ketone bodies has been shown to have multiple beneficial effects on both health and disease. For example, ketone bodies serve as an alternative fuel in trained athletes [8] and were reported to be beneficial in animal models and patients with heart failure [4,10,11,12,13]. Additionally, ketone body infusion specifically improved cognitive parameters in patients with type 2 diabetes [14]. Moreover, observations in diabetes therapy with sodium-glucose co-transporter-2 (SGLT2) inhibitor drugs suggested a positive link between beneficial heart failure outcomes and increased levels of ketone bodies [7]. It has been suggested that the beneficial effects of increased myocardial ketone body utilization are attributable to ketone bodies being used at the expense of FA, thereby leading to more oxygen-efficient oxidation [15,16], but evidence for this is lacking. More generally, the beneficial effects are related to ketones being used as a surplus fuel [17]. On the other hand, excessive levels of blood ketone bodies can lead to acidosis (ketoacidosis) [18,19]. Prolonged exposure to ketone bodies (i.e., 3HB) diminished insulin-stimulated glucose uptake in adult rat cardiomyocytes (aRCMs,) and isolated mouse soleus in association thereby suggesting inhibition of insulin signaling [20,21]. Taken together, it currently remains unclear whether changes in myocardial ketone body utilization are beneficial or detrimental for cardiac metabolism and function. These various considerations prompted us to test the effects of ketone bodies on FA and glucose handling by cardiomyocytes.

Recently, we showed that the endosomal proton pump vacuolar H+-ATPase (v-ATPase) functions as a master regulator of cardiac metabolism. V-ATPase is a multimeric protein complex, composed of an ATP-hydrolytic sub-complex (V1) and a proton translocation sub-complex (V0), and operates by a rotary mechanism [22] so as to maintain endosomal acidification [23]. We discovered that v-ATPase activity in the heart is regulated via cycles of assembly/disassembly of these sub-complexes, which can be modulated by various metabolic substrates such as FA [24], glucose [25], and AA [26]. For instance, excess lipid (e.g., palmitate) exposure of cardiomyocytes induces disassembly of v-ATPase, which impairs its function, results in loss of endosomal acidification, and subsequently triggers increased translocation of the membrane FA transporter CD36 from the endosomes to the sarcolemma. The latter initiates a vicious cycle of increased FA uptake and lipid-induced insulin resistance, eventually contributing to cardiac dysfunction [24]. In contrast, the re-assembly of v-ATPase, which was achieved through either increased glucose availability [25] or the addition of AA (in particular a mixture of lysine/leucine/arginine; 4*KLR) [26], restores lipid-induced insulin resistance and lipid-induced contractile dysfunction. Apart from the effect of palmitate, glucose, and AA, possible regulation of v-ATPase by other metabolic substrates such as ketone bodies has not been studied yet. This is especially important since there is still uncertainty about the underlying mechanisms concerning competition of ketones with other substrates and concerning their (beneficial) effects on the heart. Hence, it would be of high interest to test whether ketone bodies have an impact on the functioning of v-ATPase, either directly or indirectly.

In this study, we first tested the effects of the predominant circulating ketone bodies, acetoacetate (AcAc) and 3-β-hydroxybutyrate (3HB), on v-ATPase assembly and function in both control and lipid-overloaded rodent cardiomyocytes. After we found that 3HB, but not AcAc, treatment induces v-ATPase disassembly (just like excess lipid exposure), we subsequently studied whether 3HB-induced v-ATPase inhibition affects CD36-mediated FA uptake and lipid accumulation, insulin sensitivity, insulin-stimulated glucose uptake, and contractile function in a similar manner to excess lipid exposure. Additionally, we investigated functional alterations of v-ATPase in human-induced pluripotent stem cell-derived cardiomyocytes (hiPSC-CMs) subjected to excess ketone bodies. The collected data demonstrate that chronic 3HB exposure triggers CD36-mediated FA uptake, and then results in the loss of insulin sensitivity and contractile function via the inhibition of v-ATPase function. Forced assembly of v-ATPase via KLR supplementation prevents these 3HB-induced maladaptive changes.

## 2. Results

### 2.1. Chronic 3HB Exposure Causes v-ATPase Disassembly and De-Activation

**Figure 1:**
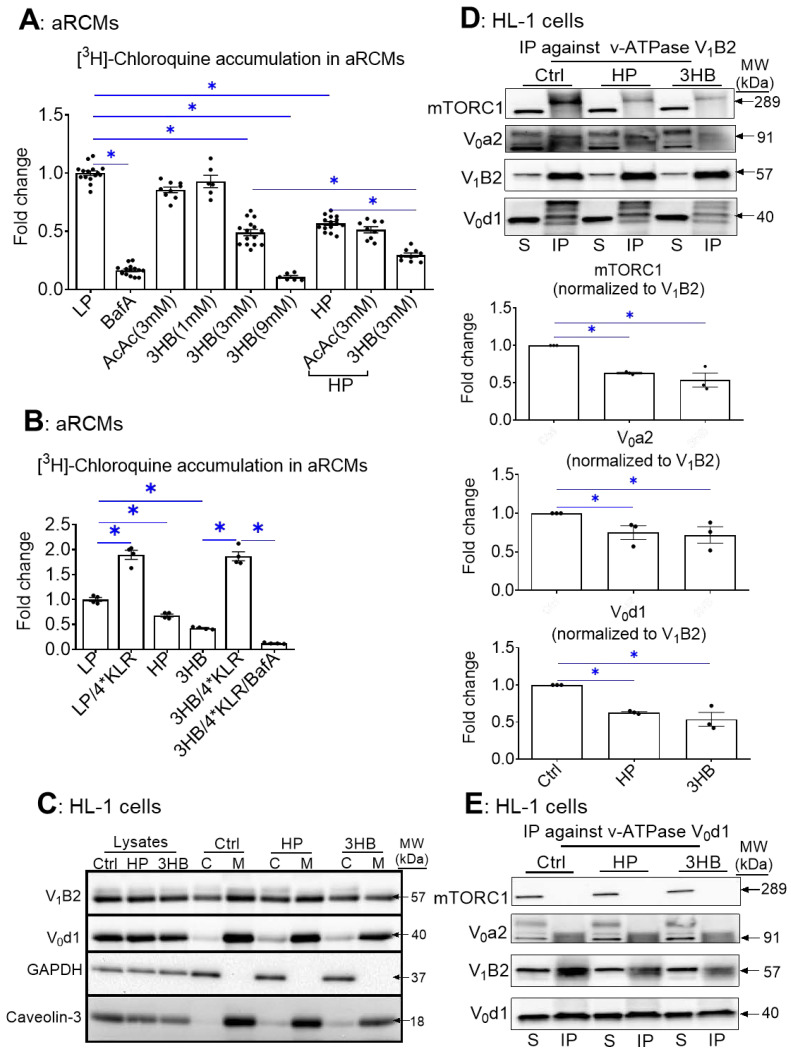

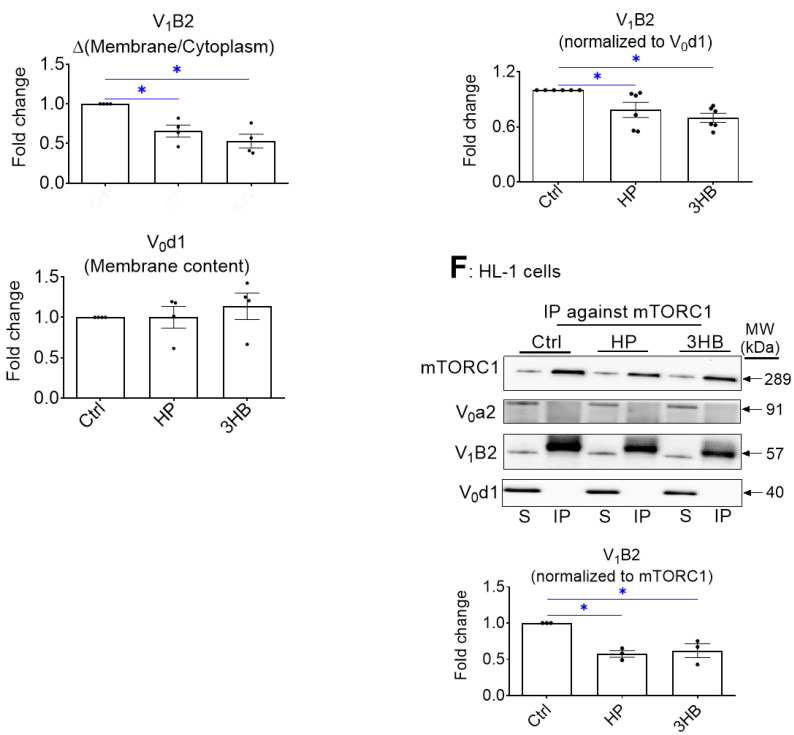
Chronic 3HB exposure causes v-ATPase disassembly and de-activation in cardiomyocytes. (**A**) 3HB exposure results in the loss of v-ATPase activity (measured as [3H]chloroquine accumulation) in cardiomyocytes: aRCMs were cultured for 24 h in either low palmitate medium (LP; control condition); LP with 100 nM bafilomycin-A (BafA), LP with 3 mM acetoacetate (AcAc); LP with different concentrations of 3-β-hydroxybutyrate (3HB; 1mM, 3 mM, and 9 mM), high palmitate medium (HP), HP with 3mM AcAc (HP/AcAc), or HP with 3mM 3HB (HP/3HB). Directly after the culturing, cells were used for the [3H]chloroquine accumulation assay. (n = 5). (**B**) Amino acids prevent 3HB-induced v-ATPase inhibition in cardiomyocytes: aRCMs were incubated for 24 h in either LP, HP, BafA, LP supplemented with 1.56 mM Lys, 1.84 mM Leu and 1.36 mM Arg, (LP/4*KLR; for explanation see Section 4.2), 3HB (3 mM), 3HB supplemented with the 4*KLR mix (3HB/4*KLR). Directly after the culturing, cells were used for the [3H]chloroquine accumulation assay (n = 5). (**C**–**F**) 3HB exposure induces v-ATPase disassembly: HL-1 cells were incubated for 24 h in either control (Ctrl) medium, HP medium, or Ctrl medium with 3 mM 3HB (3HB). (**C**) Subcellular fractionation: cytoplasmic fractions (C) and membrane fractions (M) were analyzed by Western blotting of v-ATPase subunits B2 (V1-B2) and d1 (V0-d1), after which the signals were quantified. For V1-B2, the signal ratio of membrane fraction/cytoplasm in control condition is set at 1.0. For V0-d1, the signal density in the membrane fraction is 1.0. The quantified signals in the other conditions are expressed as multiples. Representative blots are displayed. Caveolin-3 (Cav-3) and GAPDH: loading controls for membrane and cytoplasmic fraction, respectively. (n = 4). (**D**–**F**) Co-Immunoprecipitation (Co-IP) of V1, V0, and mTORC1. (**D**) IP with V1-B2, (**E**) IP with V0-d1. (**F**) IP with mTORC1. Immunoprecipitates were blotted with antibodies against mTORC1, V0-a2, V0-d1, and V1-B2, after which the signals were quantified. Representative Western blots are displayed. (n = 3). Bar values are means ± SEM. * *p* < 0.05.

First, we set out to test the effect of ketone bodies on v-ATPase activity using the [3H]chloroquine (CHLQ) method [27]. Namely, the degree of cell-associated CHLQ accumulation can be used as a readout of v-ATPase activity. Anticipating on the possible beneficial action of ketone bodies on the regulation of cardiac substrate uptake at the level of v-ATPase, we tested whether ketone bodies (AcAc and 3HB) could increase v-ATPase activity in both control and lipid-overloaded rodent adult cardiomyocytes (aRCMs). In the latter condition, the v-ATPase activity would be diminished due to the induction of v-ATPase disassembly by lipids [24]. The present results show that when compared with the low palmitate condition (LP), v-ATPase activity was reduced by 44% upon 24 h high palmitate (HP) exposure (Figure 1A), which is in line with our previous work [24,26]. Remarkably and in contrast with our hypothesis, v-ATPase activity was also reduced upon 24 h 3HB exposure (at concentrations of ≥3 mM) (i.e., >52%; Figure 1A). Additionally, 3HB further decreased v-ATPase activity on top of 24 h HP exposure. The inhibitory effect of 3HB (at 3 mM) was already for a large part present after 1 h (Supplementary Figure S1). In contrast, 3 mM AcAc had no effect (Figure 1A). Furthermore, given that we previously showed that a KLR cocktail (when each AA in this cocktail was added at 4* their concentrations as present in the portal vein of 24 h starved rats) prevented lipid-induced v-ATPase impairment [26], we also investigated whether this 4*KLR mix could prevent 3HB-induced v-ATPase impairment (Figure 1B). This was indeed the case.

Palmitate-induced v-ATPase inhibition is due to v-ATPase disassembly into its two sub-complexes [24]. Using subcellular fractionation, we verified that in all the conditions the V0-d1 subunit (as part of the membrane-bound V0 sub-complex) was detected within the membrane fraction (as was expected) (Figure 1C). Upon chronic HP and 3HB exposure, the V1-B2 subunit (as an indicator of the soluble V1 sub-complex) re-localized from the membrane fraction to the cytoplasmic fraction (Figure 1C), indicative of v-ATPase disassembly.

In a complementary approach, we studied the influence of 3HB on v-ATPase V0/V1 assembly by applying immunoprecipitation (IP) of v-ATPase sub-complexes (Figure 1D–F). Using antibodies against the d1 subunit of the V0 sub-complex (V0-d1) or against the B2 subunit as part of the soluble V1 sub-complex of v-ATPase, we verified that 3HB-rexposure leads to a lower degree of co-IP with the other subunits as compared to control condition (Figure 1D,E). Hence, these IPs confirm the findings of the fractionation: chronic 3HB exposure induces v-ATPase disassembly (Figure 1D,E). Given that lipid exposure causes the dissociation between v-ATPase and mTORC1 [26], we tested if chronic 3HB exposure would also lead to a similar dissociation of the mTOR—v-ATPase super-complex. As observed earlier [26], we found that in the LP-control condition, when v-ATPase is assembled, mTOR binds only to the V1 sub-complex, and not to V0 (Figure 1D–F). Similarly to the HP condition, 3HB exposure resulted in a reduced presence of mTORC1 in the V1B2 precipitate, and reciprocally a reduced presence of V1B2 in the mTORC1 precipitate. Therefore, similar to lipid exposure [24], 3HB exposure decreased the binding of mTORC1 to V1 (Figure 1D,F).

Taken together, these results indicate that, similarly to lipid exposure, exposure of isolated cardiomyocytes to high concentrations (3 mM and higher) of 3HB induces v-ATPase disassembly and loss of v-ATPase function.

### 2.2. Chronic 3HB Exposure Induces CD36-Mediated Lipid Accumulation

**Figure 2:**
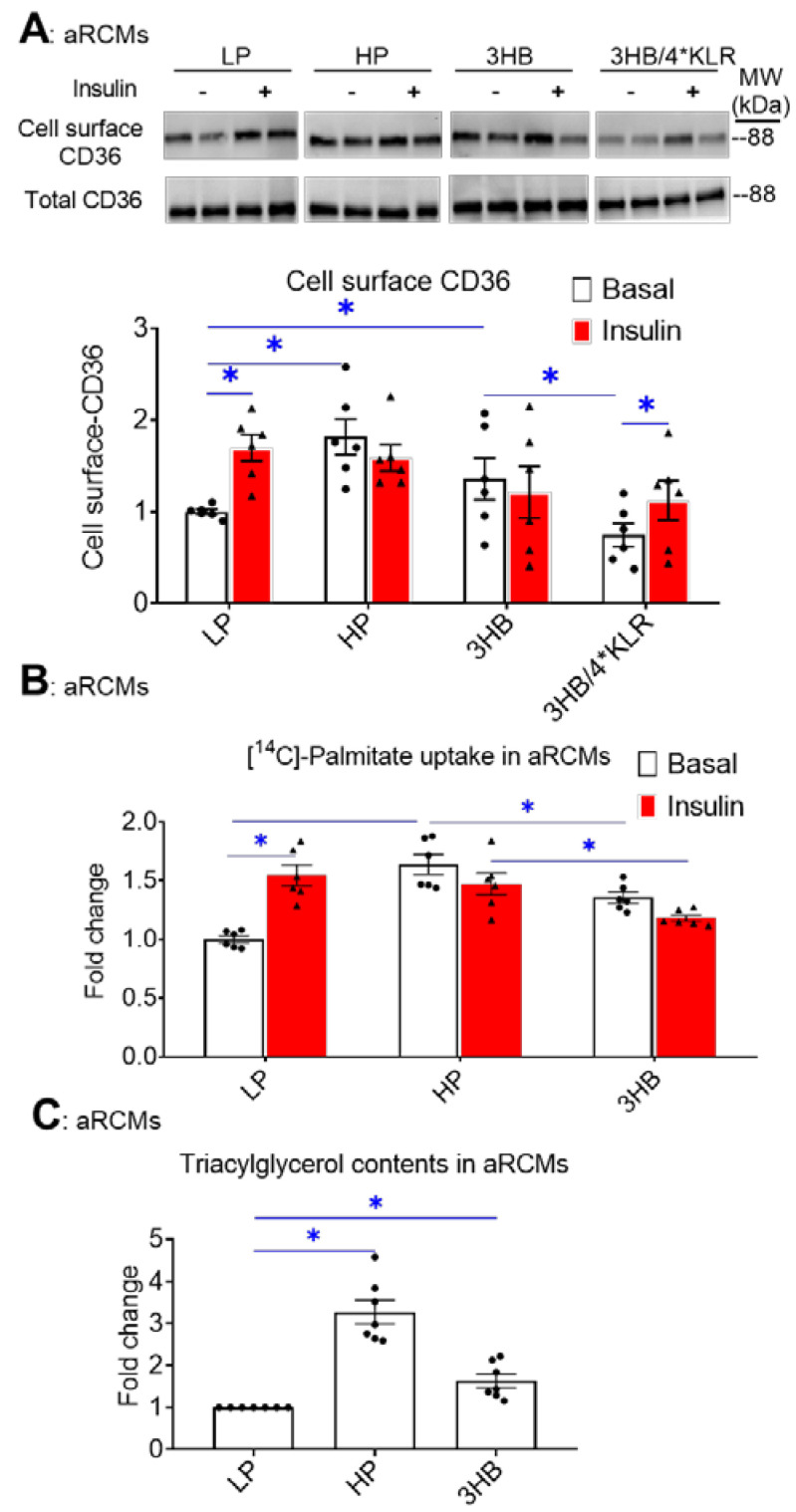
Chronic 3HB exposure induces CD36-mediated lipid accumulation in cardiomyocytes. ARCMs were cultured for 24 h in either low palmitate medium (LP; control condition), high palmitate medium (HP), LP with 3 mM 3HB (3HB), or LP with 3HB in the presence of 4*KLR (3HB/4*KLR). (**A,B**) After 24 h, cells were short-term (30 min) incubated without/with 100 nM insulin. (**A**) Assessment of cell-surface CD36 using biotinylation assay. For this, CD36 was detected by Western blotting in biotin immunoprecipitations and total cell lysates and subsequently quantified (n = 6). (**B**) [14C]palmitate uptake (n = 6). (**C**) Triacylglycerol contents (n = 7). Bar values are means ± SEM. * *p* < 0.05. The dots represent the measurements that are performed under ‘basal’ condition. The triangles represent the measurements that are performed after insulin stimulation.

In lipid-overloaded cardiomyocytes, v-ATPase inhibition leads to increased CD36 translocation to the sarcolemma, which contributes to increased FA uptake and elevated myocellular lipid levels [24]. Therefore, we investigated whether the similarity between the effects of palmitate and 3HB exposure on v-ATPase dynamics can be extended to lipid parameters. First, using a surface biotinylation assay, we observed that chronic 3HB exposure induced the translocation of CD36 to the cell surface (Figure 2A). Specifically, CD36 sarcolemmal content was increased by 1.4-fold (Figure 2A). Moreover, the 3HB-induced CD36 translocation was accompanied by a loss of short-term insulin stimulation (Figure 2A), indicating that 3HB exposure induces CD36 translocation from insulin-responsive endosomal stores. When 3HB exposure was combined with the 4*KLR mixture, 3HB-induced CD36 translocation was prevented, and insulin-stimulated CD36 translocation was improved (Figure 2A).

**Figure 3:**
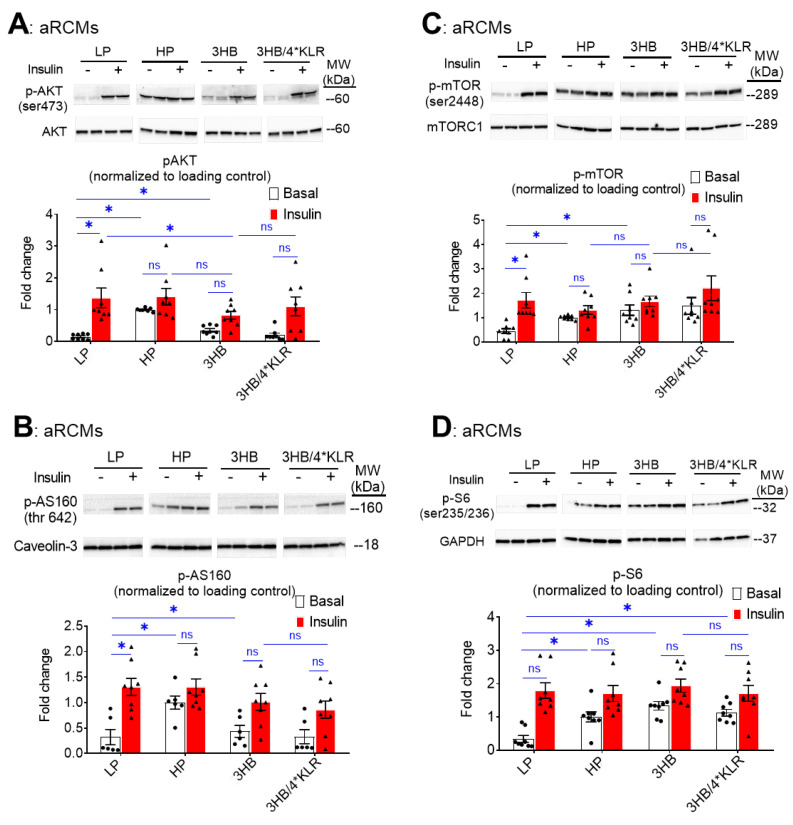
Chronic 3HB exposure interferes with insulin signaling in cardiomyocytes. ARCMs were cultured for 24 h in either low palmitate medium (LP; control condition), high palmitate medium (HP), LP with 3 mM 3HB (3HB), or LP with 3HB in the presence of 4*KLR (3HB/4*KLR). (**A**,**B**) After 24 h, cells were short-term (30 min) incubated without/with 100 nM insulin. Thereafter, (**A**) Ser473 phosphorylation of Akt, (**B**) Ser2447 phosphorylation of mTOR, (**C**) Thr 642 phosphorylation of AS160, and (**D**) Ser235/236 phosphorylation of S6 were assessed by Western blotting and quantified. For this, normalization was performed against loading controls. Loading control for the degree of phosphorylation of Akt and AS160 is caveolin-3. Loading control for mTOR and S6 phosphorylation is GAPDHs. Representative blots and corresponding loading controls are displayed (n = 8). Bar values are means ± SEM. * *p* < 0.05. ns: not significant. The dots represent the measurements that are performed under ‘basal’ condition. The triangles represent the measurements that are performed after insulin stimulation.

Next, FA uptake was studied in 3HB-exposed cells (Figure 2B). FA uptake is predominantly regulated by CD36; hence, we observed a similar pattern of changes in the FA uptake assay compared to the CD36 translocation assay: both HP exposure and 3HB caused an increase in basal FA uptake, and a loss of insulin-stimulated FA uptake (Figure 2B). Finally, the 3HB-induced increase in CD36 translocation and FA uptake in aRCMs is then expected to impact myocellular lipid levels. Indeed, myocellular triacylglycerol content was increased in cardiomyocytes upon both HP culturing (3.3-fold, Figure 2C) and 3HB culturing (1.7-fold, Figure 2C). Altogether, chronic 3HB exposure enhanced CD36-mediated lipid accumulation in aRCMs.

### 2.3. Chronic 3HB Exposure Interferes with Insulin Signaling and Insulin-Stimulated Glucose Uptake

During lipid overload, increased CD36-mediated FA uptake and lipid accumulation are known to precede the development of insulin resistance and the associated loss of insulin-stimulated glucose uptake [28]. Will the similarity between 3HB exposure and HP exposure further extend to insulin resistance? For evaluation of insulin signaling, phosphorylation levels of Akt (pAkt Ser473), mTOR (p-mTOR Ser2448), AS160 (pAS160 Thr462) and ribosomal protein S6 (pS6 Ser235/236) in aRCMs were assessed (Figure 3A–D). As expected [24], HP exposure caused a decrease in insulin-stimulated phosphorylation of each of these proteins (Figure 3A–D). Similarly to previous observations [26], the addition of the 4*KLR mixture partially prevented the 3HB-induced loss of insulin-stimulated Akt phosphorylation, and of mTOR and S6 phosphorylation. With respect to the time course of the onset of insulin resistance, it was observed that 3HB culturing induced a relatively rapid ~40% decrease in insulin-stimulated Akt/AS160/S6 phosphorylation within the first 5 h, which was followed by a phase of slower further decrease until a maximum level was reached at ~80% at 25 h (Supplementary Figure S2). Hence, 3HB and high lipid exposure of cardiomyocytes similarly decrease insulin signaling (Figure 3 and Supplementary Figure S2). Treatment of the 3HB-exposed cardiomyocytes with the 4*KLR cocktail did not prevent the negative action of 3HB on each of the phosphorylation events. Hence, at the level of insulin signaling the 4*KLR cocktail exerted no beneficial effects.

**Figure 4:**
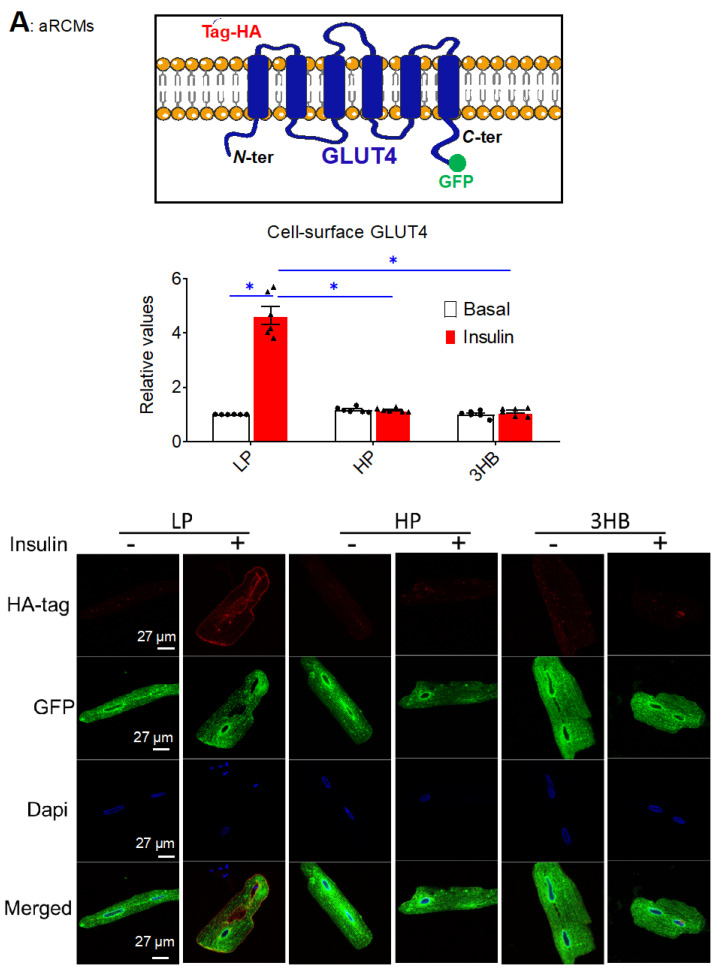

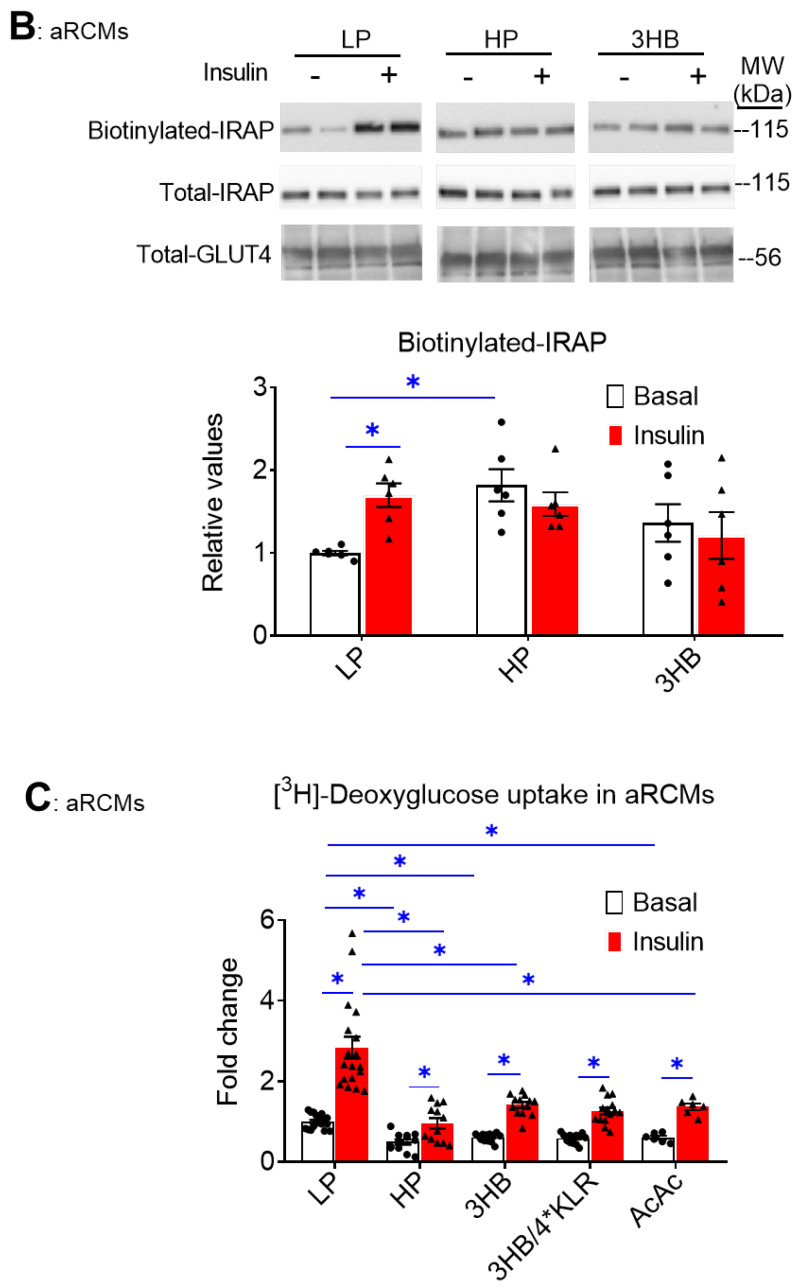
Chronic 3HB exposure prevents insulin-stimulated GLUT4 translocation and glucose uptake in cardiomyocytes. ARCMs were cultured for 24 h in either low palmitate medium (LP; control condition), high palmitate medium (HP), LP with 3 mM 3HB (3HB); LP with 3HB in the presence of 4*KLR (3HB/4*KLR); or LP with 3 mM acetoacetate (AcAc). (**A**,**B**) After 24 h, cells were short-term (30 min) incubated without/with 100 nM insulin. (**A**) Microscopical assay of cell-surface GLUT4. aRCMs were transduced for 24 h with an adenoviral vector containing an HA-GLUT4-GFP fusion protein before the start of the culturing. Non-permeabilized cells were anti-HA immunostained. The nuclei are stained in blue (DAPI). Representative microscopical images are displayed (the scale bar is 50 µm). The ratios of red (HA-tag) and green (GFP) intensity per pixel were quantified by Image J (n = 6). (**B**) Biotinylation assay. Representative Western blot and quantification of insulin-regulated aminopeptidase (IRAP, which reflects GLUT4 translocation) in biotin-immunoprecipitations and in total lysates (n = 6). (**C**) [3H]Deoxyglucose uptake (n ≥ 6). Bar values are means ± SEM. * *p* < 0.05. The dots represent the measurements that are performed under ‘basal’ condition. The triangles represent the measurements that are performed after insulin stimulation.

Next, two techniques were employed to investigate the effects of ketone bodies on insulin-stimulated GLUT4 translocation in cardiomyocytes: (i) a microscopic inspection of cell surface levels of HA-tagged GLUT4 in adenovirally transfected aRCMs (Figure 4A); (ii) a cell surface detection assay of insulin-responsive aminopeptidase (IRAP) (Figure 4B). Both methods show that insulin-stimulated GLUT4 translocation was largely decreased upon 3HB or HP exposure (Figure 4A,B), which is in line with the impairment of insulin signaling under both conditions.

Because GLUT4 translocation is a major regulatory event in cardiac glucose uptake [29], we expected that ketone bodies similarly affect glucose uptake and the influence of insulin hereon. Indeed, both lipid and 3HB exposure largely decreased insulin-stimulated glucose uptake (Figure 4C), in line with the GLUT4 translocation results. Additionally, AcAc exposure decreased insulin-stimulated glucose uptake, and the magnitude of this effect is similar to that of 3HB exposure. However, this negative AcAc action is not in line with the lack of an effect this ketone species on v-ATPase activity (Figure 1A). This discrepancy will be the topic of further discussion.

### 2.4. Chronic 3HB Exposure Induces Contractile Dysfunction

**Figure 5:**
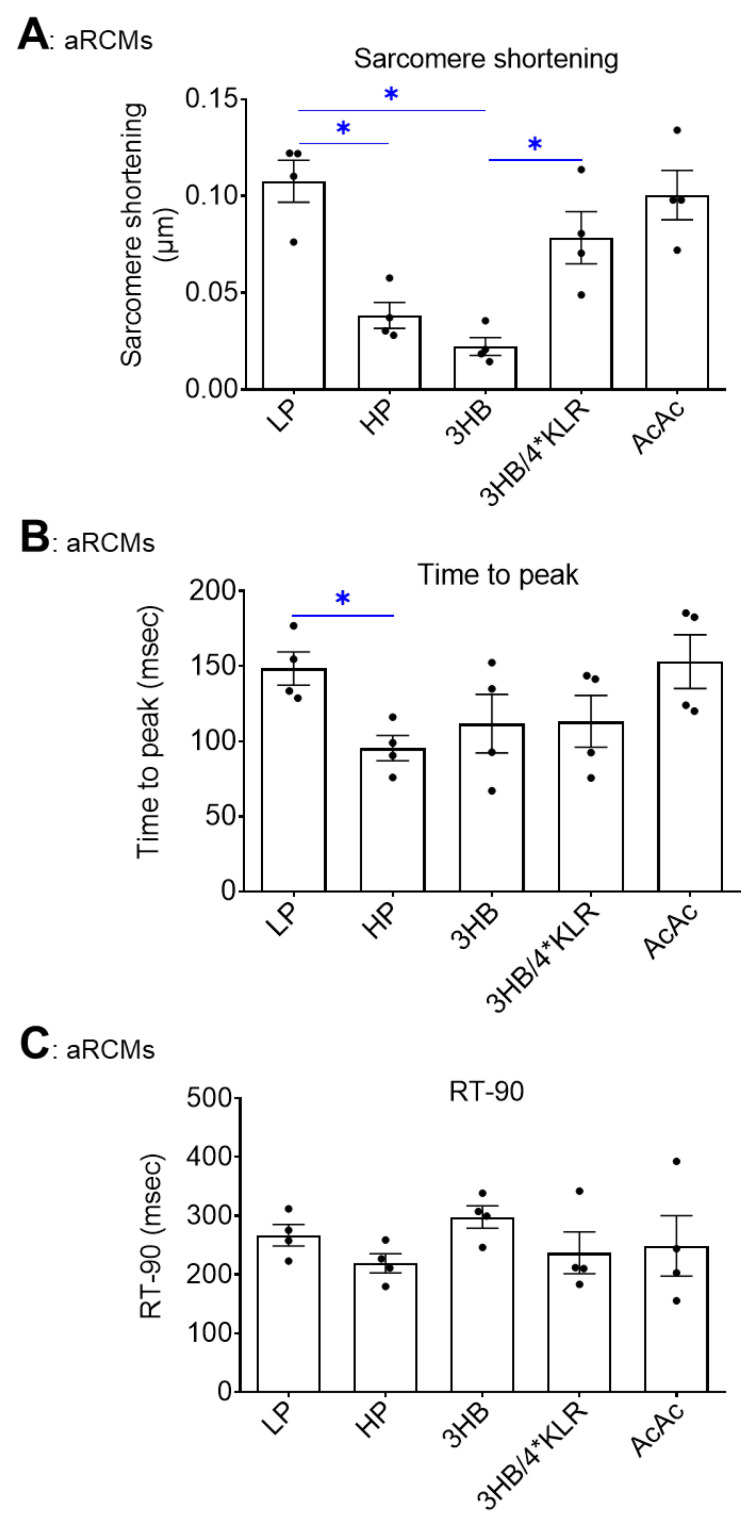
Chronic 3HB exposure induces contractile dysfunction in cardiomyocytes. ARCMs were cultured for 24 h under various conditions, being low palmitate medium (LP; control condition), high palmitate medium (HP), LP with 3 mM 3HB (3HB) or 3 mM acetoacetate (AcAc), and LP with 3HB in the presence of 4*KLR (3HB/4*KLR). The contractile parameters (**A**) sarcomere shortening, (**B**) time to peak and (**C**) decay time were deduced during electrostimulation at 1 Hz frequency (n = 4; imaging of 10 cells/measurement condition). Bar values are means ± SEM. * *p* < 0.05. The dots represent the measurements that are performed under ‘basal’ condition. The triangles represent the measurements that are performed after insulin stimulation.

As previously reported, HP exposure to cultured aRCM leads to contractile dysfunction via increased CD36-mediated myocellular lipid accumulation [30]. In this study, the negative impact of HP on contractility was confirmed (64% decrease in sarcomere shortening: Figure 5A). Additionally, 3HB exposure negatively impacted contractility, and the magnitude of the decrease in sarcomere shortening (−79%; Figure 5A) was similar to the condition of HP exposure. The other contractile parameters (time to peak, decay time) were not negatively influenced by HP or 3HB (Figure 5B,C). In contrast, AcAc exposure did not affect sarcomere shortening (Figure 5A). Finally, 4*KLR addition entirely prevented the negative effect of chronic 3HB exposure on sarcomere shortening (Figure 5A).

### 2.5. The Lipid Exposure Mimetic Effects of Ketone Bodies on v-ATPase Disassembly, Myocellular Lipid Accumulation, and Insulin Resistance Are Conserved from Rodent to Man

To examine whether the HP mimetic effect of chronic 3HB exposure on the induction of insulin resistance via v-ATPase disassembly also occurs in the human heart, hiPSC-CMs were employed. For this, hiPSCs first were characterized for their pluripotency prior to their differentiation. Pluripotent markers were highly expressed in hiPSC when compared to fibroblasts (Supplementary Figure S3A,B). Hence, these hiPSCs were pluripotent and had a strong differentiation potential at the start of their differentiation into cardiomyocytes.

**Figure 6:**
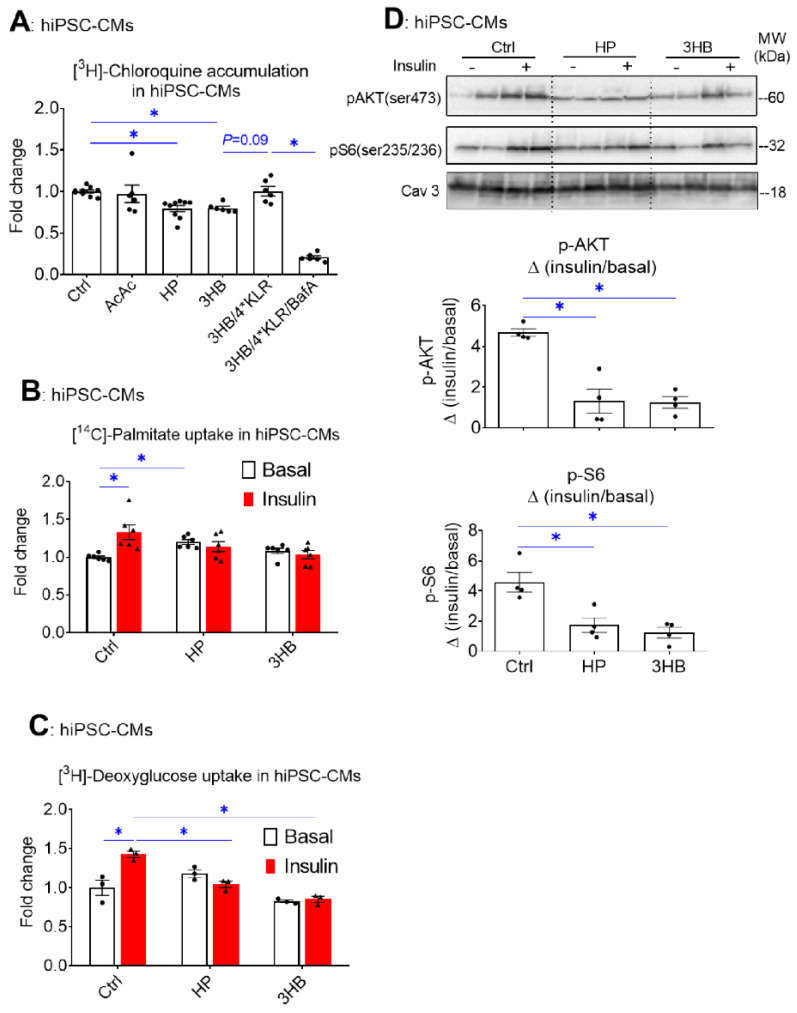
Chronic 3HB exposure reduces v-ATPase activity and insulin sensitivity in human cardiomyocytes. HiPSC-CMs were cultured for 24 h in either control medium (Ctrl), high palmitate medium (HP), Ctrl with 3mM Acetoacetate (AcAc), Ctrl with 3mM 3-β-hydroxybutyrate (3HB), 3HB with supplementation of the 4*KLR mixture (3HB/4*KLR), and with 100 nM Baf (3HB/4*KLR/BafA). (**A**) V-ATPase function in hiPSC-CMs: after culturing, cells were subjected to the [3H] CHLQ accumulation assay (n = 9). (**B**,**C**) After 24 h, cells were short-term (30 min) incubated without/with 200 nM insulin. (**B**) [14C]palmitate uptake (n = 6). (**C**) [3H] deoxyglucose uptake (n = 3). (**D**) Insulin signaling: Western analysis of phosphorylation of Akt and S6 at Ser473 and Ser235/236, respectively. Representative blots of pAKT and pS6 and Caveolin-3 (loading control) are displayed (n = 4). Bar values are means ± SEM. * *p* < 0.05. The dots represent the measurements that are performed under ‘basal’ condition. The triangles represent the measurements that are performed after insulin stimulation.

Similar to our observations in rodent cardiomyocytes (Figure 1), hiPSC-CMs exposed to HP or to 3HB displayed loss of v-ATPase function, while AcAc had no effect (Figure 6A). Moreover, hiPSC-CMs developed some key features of insulin resistance upon HP or 3HB culturing, i.e., increased basal FA uptake and loss of insulin-stimulated FA and glucose uptake (Figure 6B,C), as well as loss of insulin-stimulated Akt and S6 phosphorylation (Figure 6D). When 3HB exposure was combined with the 4*KLR mixture, the v-ATPase function was partially restored (Figure 6A), further supporting our findings in aRCMs that the addition of AA preserves insulin sensitivity via v-ATPase assembly. Taken together, the molecular mechanism of 3HB exposure-driven v-ATPase disassembly to disturb substrate uptake and impair insulin signaling in cardiomyocytes appears conserved between rodents and humans.

## 3. Discussion

Ketone bodies have been reported to exert beneficial actions in rodents and humans with heart failure [4,7,8,10,11,12,13,14,15,16]. On the other hand, ketones can induce myocellular insulin resistance [17,18,19]. These contradictory findings prompted us to assess the effects of ketones on v-ATPase, a novel key regulator of cardiac metabolism, which activity is lowered in the lipid-overloaded diabetic heart. The novel findings of the present study are that chronic exposure of cardiomyocytes to high concentrations of 3HB (3 mM or more) promotes the disassembly and de-activation of endosomal v-ATPase resulting in increased CD36-mediated FA uptake and myocellular lipid accumulation as also seen during chronic overexposure to lipids. This is followed by progressive insulin resistance and contractile dysfunction. Except for insulin-stimulated glucose uptake, these maladaptive 3HB-induced alterations are prevented by forced v-ATPase assembly via AA supplementation (4*KLR mixture). Below, we first discuss the effects of ketone bodies in the cellular context, before turning attention to the wider context of the reported beneficial actions of ketone body supplementation to elite athletes and heart failure patients.

As established earlier, HP exposure increases CD36-mediated lipid metabolism progressively, thereby contributing to the loss of insulin resistance and contractile function [24]. In this study, we show that similarly to lipids, 3HB exposure accelerates CD36-mediated FA uptake and also induces insulin resistance and contractile dysfunction. A causal relationship between altered CD36 dynamics and insulin resistance/contractile dysfunction is to be expected since CD36 deletion during lipid overload abrogates the latter two maladaptive processes [24,28,30]. The results from this study are consistent with prior studies showing that prolonged exposure to 3HB diminishes insulin-stimulated glucose uptake in aRCMs [21] and isolated mouse soleus [20], which was hypothesized to be due to the inhibition of Akt2 phosphorylation. Yet, in these studies, the effect of ketone bodies to increase lipid uptake, thus causing the observed insulin resistance, was not recognized.

In the present study, we established that the point of convergence of the maladaptive pathways of 3HB exposure and HP exposure leading to insulin resistance and contractile dysfunction involves the disassembly of v-ATPase. However, the upstream mechanism by which 3HB induce v-ATPase disassembly remains unknown, as is also the case for palmitate-induced v-ATPase disassembly [24]. Given that the other abundant ketone body, AcAc, does not affect v-ATPase function, and given that conversion of 3HB to AcAc presents the first step in ketone body oxidation, it can be assumed that ketone body metabolism and metabolites are not involved in v-ATPase disassembly. Perhaps the following two mechanisms may explain 3HB-induced v-ATPase disassembly: (i) a signaling action of 3HB directly via a cell surface receptor without having been taken up. In this respect, 3HB has been reported to be a ligand for several G protein-coupled receptors, including GPR109A and GPR41 [4]. (ii) A post-translational protein modification, β-hydroxybutyration at lysines [31]. Yet, little is known about the cellular consequences of this protein modification.

A further comparison of the effects of 3HB and AcAc on parameters of substrate uptake yields additional clues on the mechanism by which 3HB impacts contractile function. In this respect, 3HB exposure inhibits both v-ATPase assembly/function and insulin-stimulated glucose uptake. However, AcAc negatively impacts only insulin-stimulated glucose uptake and not on v-ATPase function. In combination with the observation that the degree of inhibition of insulin-stimulated glucose uptake by AcAc or by 3HB is remarkably similar (Figure 4C), this suggests that the effect of 3HB on insulin-stimulated glucose uptake is independent of that of inhibition of v-ATPase assembly/function. Hence, 3HB appears to interfere with cardiomyocyte insulin sensitivity not only at the level of v-ATPase assembly (causing CD36-mediated lipid accumulation and lipid-induced insulin resistance) but also at an additional level, which could be direct inhibition of insulin signaling, most likely at the level of Akt2, as suggested previously [20,21]. Finally, with respect to cardiomyocyte contractile activity, 3HB, but not AcAc, negatively impacts this functional parameter. This implies that the inhibition of insulin-stimulated glucose uptake (as induced by both 3HB and AcAc) is, by itself, not sufficient for inducing contractile dysfunction. Perhaps, lipid actions other than lipid-induced insulin resistance may also contribute to lipid-induced contractile dysfunction [32]. Indeed, 3HB increases myocellular lipid accumulation via v-ATPase disassembly induced CD36 translocation, whereas AcAc is unlikely to cause lipid accumulation since not inducing v-ATPase disassembly.

Another important finding of the present study is that 4*KLR counteracted the negative effects of 3HB on cardiomyocytes’ contractile function. This finding is consistent with our previous observations in lipid-overexposed cardiomyocytes [26], in which this 4*KLR mixture was employed to induce v-ATPase re-assembly in an mTORC1-dependent manner and was found to protect the cardiomyocytes from lipid-induced insulin resistance and contractile dysfunction. Hence, HP exposure and 3HB exposure not only induce similar lipid-mediated mechanisms, including v-ATPase disassembly but additionally both detrimental conditions can be improved by targeting these same mechanisms via 4*KLR treatment. Therefore, we expect that also in a combined HP + 3HB environment this specific AA mixture will be similarly beneficial which would hint toward a translational perspective. In more detail, in both conditions, the beneficial effects of the 4*KLR treatment start with the activation of mTORC1, which subsequently causes the re-assembly of v-ATPase, as established recently [26]. The subsequent endosomal acidification repurposes the endosomes as a CD36 storage compartment. As a consequence, CD36 will be re-internalized and lipid accumulation will be reversed. Finally, the lipid-induced contractile function will be relieved. However, there is one remarkable difference in the action spectrum of the 4*KLR cocktail in cardiomyocytes exposed to 3HB versus cardiomyocytes exposed to HP. The KLR cocktail prevents the loss of insulin-stimulated glucose uptake in HP-exposed cardiomyocytes, but not in 3HB-exposed cardiomyocytes. This observation provides additional evidence that 3HB impacts insulin sensitivity in cardiomyocytes not only at the level of vATPase activity but independently also at the level of Akt2 activity. The 4*KLR cocktail would then repair the v-ATPase assembly/activity via the above mechanism starting with mTORC1 activation, but not repair the second action on Akt2 inhibition. However, given that the inhibition of Akt2 in itself (i.e., by AcAc and resulting in inhibition of insulin-stimulated glucose uptake; Figure 4) is not sufficient for inducing or maintaining contractile dysfunction (i.e., AcAc does not impair contractile dysfunction; Figure 5), the inability of the 4*KLR mixture to repair this signaling step in 3HB-exposed cardiomyocytes would offer not an obstacle to still repair the contractile dysfunction via decreasing CD36-mediated lipid accumulation.

### Final Remarks

Emerging evidence suggests that ketone bodies are efficient and rapid fuels of cardiac muscle so increased cardiac uptake of ketone bodies may be an adaptive and compensatory response to the impaired energy metabolism of the diabetic or failing heart [5,10,11,33,34].

In the present study, chronic exposure of cardiomyocytes to ketone bodies resulted in the development of insulin resistance and contractile dysfunction in cultures of aRCMs. Thus, the present finding prompts us to consider why 3HB can be beneficial (as an energy substrate) as well as maladaptive (inducing insulin resistance).

In general, many cardiac diseases show a shift from the optimal substrate balance as displayed in the healthy heart, being ~60% FA, >35% glucose, and <5% alternative substrates including ketone bodies. A deviation of cardiac substrate uptake in the direction of either FA or glucose is associated with cardiac disease [35]. In the context of the diabetic heart, already characterized by increased FA uptake, the use of ketone bodies could be harmful because of the aggravation of myocellular insulin resistance. Hence, for patients with diabetic cardiomyopathy, ketone body supplementation may not be recommendable. On the other hand, patients having cardiac diseases that are accompanied by increased glucose uptake, such as pressure overload-induced cardiac hypertrophy, might benefit from ketone body supplementation. Namely, via 3HB-induced v-ATPase disassembly, the heart may be reset to increase FA uptake and hence to normalize the substrate balance. In agreement with this, 3HB supplementation proved to be beneficial in rodent models of heart failure induced by transverse aortic constriction or by myocardial infarction [11,13]. Both preclinical heart failure models would be expected to shift cardiac substrate preference towards glucose [36]. With respect to elite athletes, these individuals are often supplemented with ketone-containing drinks to enhance their sporting performance. Ketone supplementation is expected not to be harmful to this specific group, because these individuals are likely to be extremely insulin sensitive and 3HB may thus not be capable of reducing insulin signaling to a worrying extent, especially if compensated by immediate exercise. Finally, for patients with late-stage heart failure in which both FA uptake and glucose uptake have collapsed [37], the supplementation of ketone bodies as alternative substrate might provide the last resort to generate energy. In conclusion, it may depend very much on the metabolic status of the heart, whether ketone body supplementation should be considered potentially harmful or beneficial. In future in vivo studies, it should be re-assessed whether 3HB supplementation to rodents would cause lipid-induced cardiomyopathy.

## 4. Materials and Methods

### 4.1. Antibodies

Details on antibodies that are used for Western blotting and immunofluorescence (IF) analysis are provided in Supplementary Table S1.

### 4.2. Isolation and Culturing of Primary Rat Cardiomyocytes

Male Lewis rats, 250–300 g, were purchased from Charles River laboratories, and were maintained at the Experimental Animal Facility of Maastricht University. Animals were housed in a controlled environment (21–22 °C) on a 12:12 h light dark cycle (light from 0700 to 1900 h) and had free-access to food and tap water.

Adult rat cardiomyocytes (aRCMs) were isolated by using a Langendorff perfusion system, as previously described [38]. Briefly, after a 2 h adhesion period, aRCMs were incubated for 24 h in either low palmitate (LP, palmitate/BSA ratio 0.3:1), LP supplemented with 100 nM of v-ATPase inhibitor Bafilomycin-A (BafA), LP supplemented with 3 mM AcAc, LP supplemented with different concentrations of 3HB (1 mM, 3 mM, and 9 mM), 3HB supplemented with the mixture of 4*KLR (Arg-1.36 mM, Leu-1.84 mM and Lys-1.56 mM, AA concentration is 4 times higher in M199 medium) [3HB/4*KLR], high palmitate (HP, palmitate/BSA ratio 3:1), HP supplemented with 3 mM AcAc (HP/AcAc), or HP supplemented with 3 mM 3HB (HP/3HB).

### 4.3. Culturing of HL-1 Cardiomyocytes

HL-1 cells were kindly provided by Dr. W. Claycomb (Louisiana State University, New Orleans, LA, USA) and cultured as previously described [24]. Briefly, HL-1 cells were cultured for 24 h in either control medium (Ctrl; no palmitate), Ctrl supplemented with 3 mM 3HB, or high palmitate medium containing 500 µM palmitate (palmitate/BSA ratio 6:1) and 100 nM insulin (HP).

### 4.4. Human Induced Pluripotent Stem Cell (hiPSC) Maintenance and Differentiation into Cardiomyocytes (hiPSC-CMs)

Skin fibroblasts from healthy adult male individuals were collected and hiPSCs were generated by episomal reprogramming at the Stem Cell Technology Centre, Radboudumc (Nijmegen, The Netherlands). The cells were maintained in Essential 8 medium (Thermofisher Scientific, Miami, FL, USA) under feeder-free conditions. Prior to cardiomyocyte differentiation, the cells were passaged with 0.5 mM of EDTA solution (Promega, Madison, WI, USA), counted with a cell-counter and seeded in Essential 8 medium containing 10 µM ROCK inhibitor (Stem Cell Technologies, Vancouver, BC, Canada) on Matrigel (Corning Inc., NY, USA)-coated 24-well and 12-well plates. This was denoted as day 4 and the medium was changed daily until the cells reached 80%–90% confluency. At day 1 of differentiation, Essential 8 medium was removed and replaced with cardiomyocyte medium a (Thermofisher Scientific, Miami, FL, USA). The cells were incubated for 2 days prior to a media change to cardiomyocyte medium b (Thermofisher Scientific, Miami, FL, USA). At day 5, the media was then replaced with cardiomyocyte maintenance media (Thermofisher Scientific, Miami, FL, USA) for 2 days and replaced every 2 days until day 14. To purify the cardiomyocyte popultion, metabolic selection was performed using RPMI 1640 without glucose (Thermofisher Scientific, Miami, FL, USA) containing 4 mM sodium lactate (Sigma Aldrich, St. Louis, MO, USA) for 5 days and thereafter the cells were further maintained in cardiomyocyte maintenance media for an additional 5 days. Finally, the metabolically selected hiPSC-CMs were cultured for 24 h in the same Ctrl/HP/3HB-containing media as described for the HL-1 cells.

### 4.5. Measurement of v-ATPase Disassembly/Assembly

Two methods were applied to measure disassembly, namely, immunoprecipitation (IP) (i) and subcellular fractionation (ii).

(i) IP: Upon termination of culturing, cardiomyocytes were washed with ice-cold PBS twice and lysed with lysis buffer containing 1% Brij O20, 250 mM NaCl, 5 mM EDTA and 50 mM HEPES (pH 7.0). An amount of 500 µg cell suspension was incubated overnight at 4 °C with control IgG or specific antibodies recognizing v-ATPase B2 or a2 subunits. Then, the protein–antibody complex was coupled to Sepharose-4 beads for 4 h at 4 °C. Beads were washed 5x in lysis buffer, boiled in sample buffer (125 mM Tris-HCI, pH 6.8, 2% SDS, 10% sucrose, 50 mM dithiothreitol, and 0.01% bromphenol blue), centrifuged, after which the supernatant was used for Western blotting. v-ATPase a2 (V0-a2), v-ATPase d1 (V0-d1, an indicator of the membrane-bound V0 sub-complex), v-ATPase B2 (V1-B2, an indicator of cytosolic V1 sub-complex), and mTORC1 proteins were detected by Western blotting.
(ii) Subcellular fractionation: Upon termination of culturing, the cells were washed twice with ice cold PBS and lysed using SET Buffer 10 mM Tris HCl, pH 7.4, 0.25 M sucrose, 2 mM EDTA, and Complete Protease Inhibitor Cocktail (Roche)). Cells were subjected to 3× freeze–thawing cycles in liquid nitrogen and spun down at 500× *g* for 1 min to get rid of the debris. Cells were subsequently centrifuge at 200.000× *g* for 1 h using Beckman Coulter OptimaTm Max-xp ultracentrifuge 200.000× *g* Rotor TLA-100 (SN440) to separate membrane and cytosolic fractions. After centrifugation, supernatant (cytosol fraction) was stored, and the remaining pellet (membrane fraction) was dissolved in 200 uL SET Buffer. Both fractions were stored at −80 °C, and later used for Western detection of V0-d1, V1-B2, GAPDH, and caveolin-3 (Cav-3).

### 4.6. Measurement of Cellular Chloroquine (CHLQ) Accumulation as Readout of v-ATPase Function

[3H] CHLQ accumulation assay in HL-1 cells, aRCMs, and hiPSC-CMs was measured as follows. At the end of the culturing, cells were incubated for 20 min with 0.46 uL/well [3H]chloroquine (1 µCi/uL diluted 1:5 in Milli-Q). Then, cells were quickly washed with ice-cold PBS and subsequently scraped from the well using NaOH. Lysates were transferred to vials containing 5 mL Opti-Fluor (Perkin Elmer, Waltham, MA, USA) Samples were counted with a (Wallace?) liquid scintillation counter.

### 4.7. Surface-Protein Biotinylation for GLUT4 and CD36

Surface-protein biotinylation was measured as previously described with the modifications described below [25]. After culturing, aRCMs were incubated for 30 min (−/+) 100 nM insulin. Subsequently the cells were biotinylated with the cell-impermeable reagent sulfo-NHS-LC-biotin (0.5 mg/mL dissolved in M199 medium, Thermo Fisher Scientific, Fremont, CA, USA) for 10 min at 37 °C. Cells were subsequently washed with ice-cold glycine in DPBS (7.5 mg/mL), to quench and remove the excess of biotin, and finally lysate with lysis buffer. Lysates were incubated on ice for 10 min., and centrifugated 15 min at 4 °C in the presence of streptavidin beads. Next day samples were centrifugated and streptavidin beads washed with lysis buffer. Biotinylated proteins were eluted from beads using sample buffer for 5 min at 95 °C. Insulin-regulated aminopeptidase protein (IRAP, which reflects GLUT4 trafficking), and CD36 were detected by Western blotting.

### 4.8. Microscopic Assessment of GLUT4 Translocation

To further assess GLUT4 translocation, adenoviral constructs expressing hemagglutinin (HA)-GLUT4-green fluorescent protein (GFP) (a gift from Prof. Luc Bertrand’s lab, Institute of Experimental and Clinical Research (IREC), Pole of Cardiovascular Research, UCLouvain, Brussels, Belgium) were employed in this study. Evaluation of GLUT4 translocation was conducted as previously described with the modifications described below [39]. Briefly, GFP was fused to the carboxyl terminus of GLUT4 to track all exogenous GLUT4. In contrast, HA epitope was inserted in the first exofacial loop of GLUT4, allowing the exclusive detection of GLUT4 inserted into the membrane of non-permeabilized cells. aRCMs were infected with HA-GLUT4-GFP adenoviruses at a multiplicity of infection of 5 for 45 h. After paraformaldehyde 4% (vol/vol) fixation and blocking with BSA 5% (wt/vol), non-permeabilized cells were incubated with a primary antibody (anti-HA), followed by secondary fluorescent antibody (Alexa Fluor 594). Nuclei were stained with DAPI. The cells were mounted on a glass slide and imaged at 63x objective with the confocal microscope (Leica SPE). Red fluorescent dots (HA staining) were quantified relative to GFP by ImageJ Fiji, version 1.45d.

### 4.9. Quantification of Triacylglycerol Contents

Quantification of triacylglycerol in aRCMs was performed using a Triglyceride Assay Kit (ab65336, Abcam, San Francisco, CA, USA) following the manufacturer’s instructions.

### 4.10. Determination of Insulin Signaling

Insulin signaling in aRCMs were measured as previously described [25]. Briefly, after culturing, aRCMs were exposed to 100 nM insulin for 30 min to be able to compare basal phosphorylation to insulin-stimulated phosphorylation. Afterward, these cells were lysed in sample buffer and used for protein detection by SDS-polyacrylamide gel electrophoresis, followed by Western blotting. Phospho-Ser2448-mTOR (p-mTOR), phospho-Ser473-Akt (pAKT), phospho-AS160, phospho-Ser235/236-S6 (pS6), total-mTOR, total-AKT, GAPDH, and caveolin-3 proteins were detected by Western blotting.

### 4.11. Measurement of Substrate Uptake

[3H]deoxyglucose and [14C]palmitate uptake in aRCMs and hiPSC-CMs were measured as follows. Palmitate (coupled to BSA in a palmitate/BSA ratio of 1:3) and deoxy-D-glucose were added to the cell cultures to final concentrations of 20 and 4 μmol/L, respectively, with tracer amounts of [14C]palmitate and 2-deoxy-D-[3H]glucose. After 10 min, uptake was terminated, and unbound substrate removed by washing the cells with ice-cold depletion medium containing 0.2 mmol/L phloretin. After transfer of the glass slides into new culture dishes, cells were lysed by addition of 1 mol/L NaOH. Subsequently, incorporated glucose and palmitate were measured by scintillation counting of 14C and 3H.

### 4.12. Measurement of Cardiomyocyte Contraction Dynamics

Contractile properties of aRCMs were assessed at 1 Hz field stimulation using a video-based cell geometry system to measure sarcomere dynamics (IonOptix, Milton, MA, USA). From the digitized recordings acquired with IonWizard acquisition software, the following parameters were calculated: sarcomere shortening, time to peak, and decay time.

### 4.13. Immunofluorescent Staining for Pluripotency Markers in hiPSC-CMs

Immunofluorescent staining for pluripotency markers (e.g., SSEA4 and OCT4) in hiPSC-CMs was conducted as follows Cells were fixed with fixative solution and incubated for 15 min at RT. The staining procedure was performed as per manufacturer’s instructions using the Pluripotency Stem Cell 4-Market Immunocytochemistry kit (Thermofisher Scientific). Permeabilization solution was added to the cells and incubated for 15 min at RT. Blocking solution was added and incubated for 30 min. The cells were stained with primary antibody (1:100 SSEA4 with 1:200 OCT4 in blocking buffer) for 1 h. Cells were washed with washing buffer and stained with the secondary antibody (Alexa Fluor 488 and Alexa Fluor 555) for 1 h at room temperature. Cells were washed with wash buffer and 1 drop of NucBlue Fixed Cell stain (DAPI) was incubated for 5 min. The cells were mounted on a glass slide and imaged at 63x objective with the confocal microscope (Leica SPE). Images were analyzed with ImageJ Fiji, version 1.45d.

### 4.14. QPCR Analysis for Pluripotency Markers in hiPSC-CMs

QPCR analysis was performed for several pluripotency markers (OCT4, SOX2, NANOG, and LIN28) in hiPSC-CMs. RNA of the samples was isolated using the High Pure RNA isolation kit (Roche). After RNA was extracted, cDNA synthesis was performed. The cDNA synthesis was carried out in a total volume of 20 µL containing 1× qScript cDNA supermix (Quantabio) and 500 ng RNA template. PCR program: 5 min at 25 °C, 30 min at 42 °C, 5 min at 80 °C and hold at 4 °C The qPCR was carried out in a total volume of 10 µL containing 2× Sensimix SYBR Hi-ROX (Bioline), 25 uM primers and 5 times diluted cDNA. Samples are run on the Lightcycler 480 (Roche) with the following program: 10 min at 95 °C and 40 cycling with 15 s at 95 °C, 15 s at 60 °C and 15 s at 72 °C, with the relative quantification analysis. Supplementary Table S2 displays primers for pluripotency genes in hiPSC-CMs.

### 4.15. Statistics

Each value, indicated by a dot in the bar figures, presents the mean of two or three distinct measurements with different cells cultured in separate wells within one experiment. In every single experiment, the cells are derived from one rat heart (in case of aRCM) or from one cell passage (in case of HL1 cells or hi-PSC-CMs). Hence, each dot presents one independent measurement in duplicate or triplicate. For the Western blots, these duplicates from separate wells within one experiment are on display. Statistical analyses were performed using IBM SPSS Statistics 23 (SPSS Inc., Chicago, IL, USA) and GraphPad 8.0 PRISM^®^. Briefly, the data were compared using a one-way ANOVA followed by Duncan’s post hoc tests (among the groups, i.e., different culturing conditions), or paired Student’s *t*-test (within groups, i.e., when analyzing short-term insulin effect). All data are presented as mean ± SEM. *p*-values < 0.05 are considered statistically significant.

## Data Availability

The datasets used and/or analyzed during the current study are available from the corresponding author upon reasonable request.

## Supplementary Materials

The supporting information can be downloaded at: https://www.mdpi.com/article/10.3390/ijms232112909/s1.
