# Supplementary material for: Ketone Body Exposure of Cardiomyocytes Impairs Insulin Sensitivity and Contractile Function through Vacuolar-Type H+-ATPase Disassembly—Rescue by Specific Amino Acid Supplementation
Source: Int J Mol Sci. 2022 Oct 26;23(21):12909. doi: 10.3390/ijms232112909 (PMC9657709; doi:10.3390/ijms232112909)
Supplement: Supplementary file 1 [file ijms-23-12909-s001.zip › ijms-1866131-supplementary.pdf]

Supplementary Data

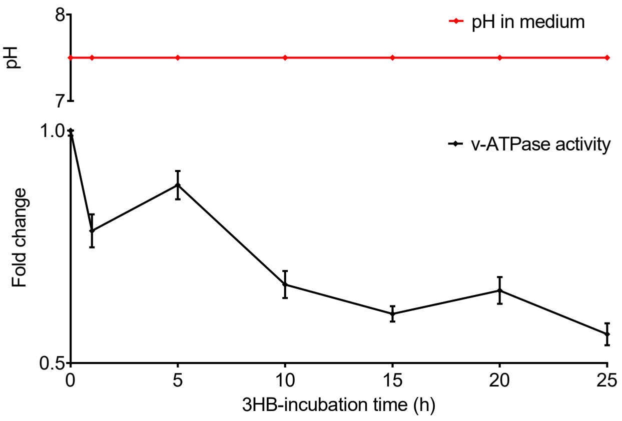

Supplemental Figure S1 (related to Figure 1A). The time course of 3HB induced-effect on v-ATPase activity in adult rat cardiomyocytes (aRCMs): aRCMs were incubated with low palmitate (LP, palmitate/BSA ratio 0.3:1) and high palmitate (HP, palmitate/BSA ratio 3:1) for 25 h, and 3 mM of 3-β-hydroxybutyrate (3HB) was added at the start of the LP culturing for 0, 1, 5, 10, 15, 20, and maximum 25 h. After the culturing, cells were used for a [3H] chloroquine (CHLQ) accumulation assay which lasted 20 min (n = 4). Values are displayed as mean ± SEM.

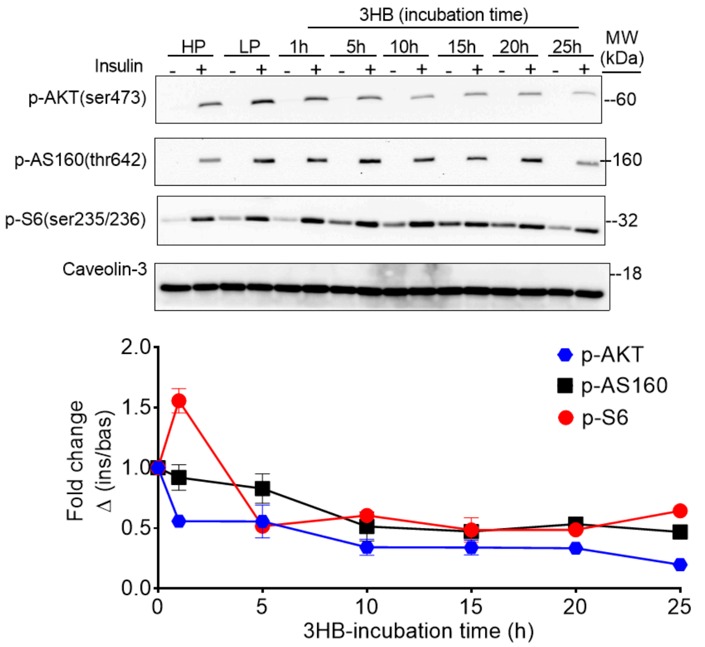

Figure S2. (Related to Figure 3). Time course of 3HB exposure interferes with insulin signaling in adult rat cardiomyocytes (aRCMs): aRCMs were incubated with low palmitate (LP, palmitate/BSA ratio 0.3:1) and high palmitate (HP, palmitate/BSA ratio 3:1) for 25 h, and 3 mM of 3-β-hydroxybutyrate (3HB) was added at the start of the LP culturing for 0, 1, 5, 10, 15, 20 and maximum 25 h. After the culturing, aRCMs were followed by 30 min of (-/+) insulin (100 nM) incubation. Representative blots and its quantification of phospho-Ser473-Akt (pAKT), phospho-AS160, phospho-Ser235/236-S6 (pS6), and Cav-3 (loading control) (n = 3). All of the quantification values normalized to the LP condition for the 0 time point. Values are displayed as mean ± SEM. * $p < 0.05$ were considered statistically significant.

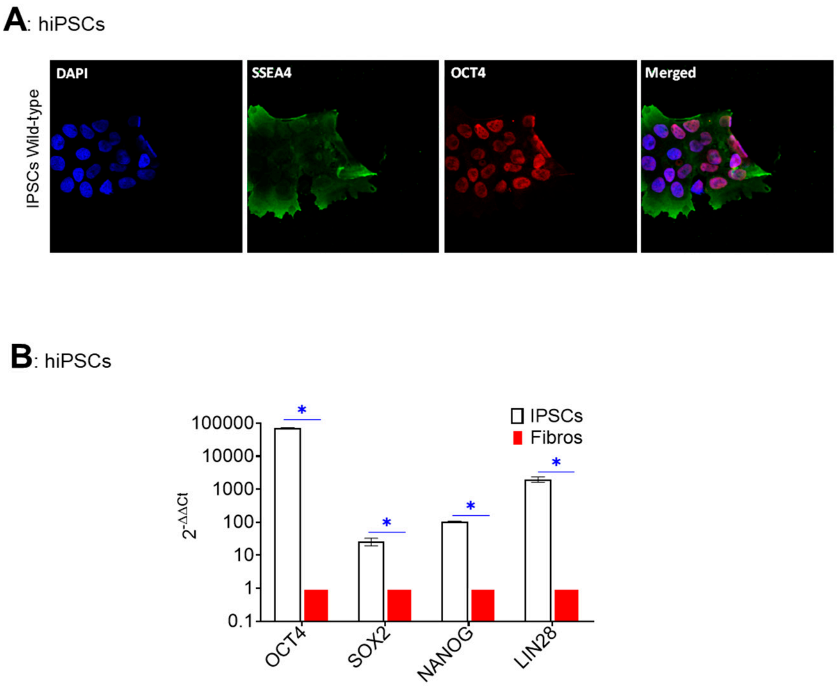

Figure S3. (Related to Figure 6). (A-B) Characteristics of hiPSCs: **(A)** Expression of pluripotency markers (e.g., SSEA4 and Oct4) and DAPI in hiPSCs, assayed by immunofluorescence microscopy. Scale bar is 10 µm. **(B)** Gene expression of pluripotency markers in hiPSCs relative to fibroblasts, as assayed by qPCR (n = 3). Bar values are means ± SEM. *$p < 0.05$ were considered statistically significant.

Table S1. Antibodies used in this study.

Antibodies	Source	Identifier
p-AKT (Ser473)	Cell signaling	#9271
Total-AKT	Cell signaling	#9272
p-mTOR (Ser2448)	Cell signaling	#2971
Total-mTOR	Cell signaling	#2972
pS6 (Ser235/236)	Cell signaling	#4856
p-AS160 (Thr462)	Cell signaling	#4288
Insulin-regulated aminopeptidase (IRAP)	Cell signaling	#MABN483
GAPDH	Cell signaling	#2118
Anti-ATP6V_0D1 (V0d1)	Abcam	#ab202899
Anti-ATP6V_1B2 (V1B2)	Abcam	#ab73404
Anti-ATP6V0A2 (V0-a2)	Abcam	#ab82638
HA-Tag (C29F4)	Abcam	#3724(S)
CD36 (MO25)	Gift from Dr.N.Tandon	No
GLUT4	Millipore	#07-1404
Caveolin-3	BD transduction Laboratories	#610421
DAPI	Sigma	#28718-90-3
SSEA4	Thermo Fisher Scientific	MA1-021
OCT4	Thermo Fisher Scientific	# MA1−104

Table S2. Primers for pluripotency genes in human induced pluripotent stem cells.

**Pluripotency genes**	**Species**	**Primer sequence**	**Melting temperature (°C)**	**Fwd/Rev**
SOX2	Human	CCCAGCAGACTTCACATGT	60	fwd
SOX2	Human	CCTCCCATTTCCCTCGTTTT	60	rev
OCT4	Human	CCTCACTTCACTGCACTCTA	60	fwd
OCT4	Human	CAGGTTTTCTTTCCCTAGCT	60	rev
NANOG	Human	CTCCATGAACATGCAACCTG	60	fwd
NANOG	Human	GGCATCATGGAAACCAGAAC	60	rev
LIN28	Human	CACAGGGAAAGCCAACCTAC	60	fwd
LIN28	Human	TGCACCCTATTCCCACTTTC	60	rev
